# A Puzzling Thank You

**DOI:** 10.3928/24748307-20221213-01

**Published:** 2023-01

**Authors:** Michael K. Paasche-Orlow

Riddle me this: What sport requires more referees than contenders? Academic journals! Thank you to all the reviewers listed below for all of your efforts this past year; the 10 highest-rated reviewers are identified with an asterisk.

Here is a puzzle of gratitude in your honor (**Figure 1**). Make a list of words using the letters in the puzzle according to the following rules: (1) each word must include the central letter; (2) words must be at least five letters long; (3) letters can be repeated; (4) proper nouns and words that are offensive or hyphenated do not count; (5) each word is worth one point—other than a “pangram,” which uses every letter at least once. A pangram is worth three points—and there is a pangram here that fits the theme of this statement of gratitude. We will make a donation to an adult literacy organization in the name of the winner! Please email me your list.

**Figure 1.**
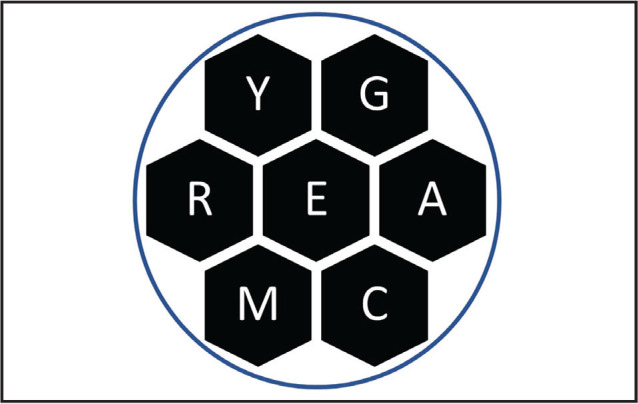
Gratitude puzzle.

## *HLRP: Health Literacy Research and Practice* Reviewers 2022

Mary Ann Abrams
Traci Alberti
Linda Aldoory
Kathryn Anderson
Melissa Anderson
Brittany Ange
Evans Ansu-Yeboah
Stacy Cooper Bailey
Thomas Bauer
Geri Baumblatt
Cindy Brach
Howard Cabral*
Elena Carbone
Enrique Castro-Sanchez
Xuewei Chen
Daniel Chu
Michael Cooper
Radhika Devraj
Darren A. Dewalt*
Gerardine Doyle
Gregory Duncan*
Iris Feinberg*
Sasha Fleary
Mirjam Fransen
Nerissa George
Alexander Glick*
Kelsey Grabeel*
Jacek Gwizdka
Eleanor Hall
Emily Hurstak*
Claudia Isonne
Kazuhiro P. Izawa
Paul Jones
Karen Komondor
In Sook Lee*
Elizabeth LeQuieu
Angela Leung
Diane Levin-Zamir
Karsten Lunze
Heather Mccormack
Cathy D. Meade
Melanie Messer
Ann Miller
Nancy Morris
Laxsini Murugesu
Danielle Muscat
Tam Nguyen
Raymond L. Ownby
Michael Paasche-Orlow
Christine Prue
Audrey Riffenburgh
Donald Rubin*
Katie Shradley
Paul Smith
Carl Streed
Kristie Weir
Barry D. Weiss*
Lauren Wheeler
Fedayi Yagar
Maryam Yuhas

